# Integrated Proteomics and Machine Learning Approach Reveals PYCR1 as a Novel Biomarker to Predict Prognosis of Sinonasal Squamous Cell Carcinoma

**DOI:** 10.3390/ijms252413234

**Published:** 2024-12-10

**Authors:** Watcharapong Panthong, Chamsai Pientong, Thawaree Nukpook, Sittiruk Roytrakul, Yodying Yingchutrakul, Watchareporn Teeramatwanich, Sirinart Aromseree, Tipaya Ekalaksananan

**Affiliations:** 1 Department of Microbiology, Faculty of Medicine, Khon Kaen University, Mueang Khon Kaen, Khon Kaen 40002, Thailand; watchara.p@kkumail.com (W.P.); thawaree.n@kkumail.com (T.N.); siri-nar@kku.ac.th (S.A.); 2 HPV&EBV and Carcinogenesis (HEC) Research Group, Faculty of Medicine, Khon Kaen University, Mueang Khon Kaen, Khon Kaen 40002, Thailand; 3 Functional Ingredients and Food Innovation Research Group, National Center for Genetic Engineering and Biotechnology, National Science and Technology Development Agency, Khlong Luang, Pathum Thani 12120, Thailand; sittiruk@biotec.or.th (S.R.); yodying.yin@nstda.or.th (Y.Y.); 4 Department of Otorhinolaryngology, Faculty of Medicine, Khon Kaen University, Mueang Khon Kaen, Khon Kaen 40002, Thailand; yui.entkku@gmail.com

**Keywords:** sinonasal squamous cell carcinoma, proteomic, machine learning, biomarker, prognosis

## Abstract

Sinonasal squamous cell carcinoma (SNSCC) is a rare tumor with a high 5-year mortality rate. However, proteomic technologies have not yet been utilized to identify SNSCC-associated proteins, which could be used as biomarkers. In this study, we aimed to discover a biomarker to predict SNSCC patients using proteomic analysis integrated with machine learning models. Support vector machine (SVM), logistic regression (LR), random forest (RF), and gradient boost (GB) classifiers were developed to predict SNSCC based on proteomic profiles of SNSCC compared with nasal polyps (NP) as control. Seventeen feature proteins were found in all models, indicating possible biomarkers for SNSCC. Analysis of gene expression across multiple cancer types and their associations with cancer stage and patient survival in the TCGA-HNSC dataset identified a PYCR1 and MYO1B gene that could be a potential tumor-associated marker. The expression of PYCR1 was confirmed by RT-qPCR in SNSCC tissues, and its high expression was associated with poor overall survival, indicating PYCR1 as a potential tumor-associated biomarker to predict the prognosis of SNSCC.

## 1. Introduction

Nasal cavity cancers represent 5% of all head and neck cancers and have a histological variety and complexity, which is a challenge for pathologists. In general, patients usually have an asymptomatic appearance until the tumor grows to a large size. Therefore, most of the patients were found at an advanced stage [1]. The most common type of nasal cancer is sinonasal squamous cell carcinoma (SNSCC), which is approximately 50% of all nasal cancer. SNSCC mainly originates in the nasal cavity and maxillary sinus. The incidence of SNSCC is <1 case per 100,000 population, which is found in men more than women. The majority of patients are aged 50–60 years old and have a 53.1% 5-year survival rate [1,2].

Recently, most researchers have focused on genetic mutation and environmental factors that influence SNSCC. The environment-associated risk factors of SNSCC are woodworkers, occupational exposure to chemical substances, and the leather industry [3]. These risk factors are possibly involved in the chronic inflammation pathway [2]. EGFR and KRAS mutation have been linked to be associated with SNSCC development [4]. Nevertheless, little is currently known about candidate protein biomarkers for SNSCC. Therefore, exploring a novel biomarker is critical for the diagnosis and prognostic prediction of SNSCC patients.

In the last decade, proteomic analysis has become a promising tool for the study of tumor biology. The purpose of clinical proteomics studies is to identify diagnostic biomarkers, understand the molecular pathogenesis of cancers, identify drug targets, and personalize medicine [5]. Recently, machine learning (ML) has enabled biologists to uncover the underlying biology of large-scale omics datasets and shown promise in improving diagnosis, and predicting risk of diseases based on various factors such as clinical information, biochemical testing, electrocardiograms, medical imaging, and biomarkers [6,7,8,9]. These techniques can be used to support physicians and scientists in studying and classifying anatomic pathologies. In the context of SNSCC, a rare and aggressive head and neck cancer, identifying robust biomarkers is crucial for early diagnosis, personalized treatment, prognostic prediction, and patient outcome improvement. Therefore, we aimed to identify potential tumor-associated markers through proteomic analysis integrated with ML models. Our findings suggest that a combination of ML and proteomic data can be used to classify SNSCC and nasal polyps (NP) patients. Specifically, we observed for the first time that PYCR1, a gene involved in proline metabolism, exhibits promise as a tumor-associated marker and can be significantly used as a prognostic biomarker in SNSCC.

## 2. Results

### 2.1. Label-Free Quantification of Nasal Polyps and Sinonasal Squamous Cell Carcinoma

**Figure 1:**
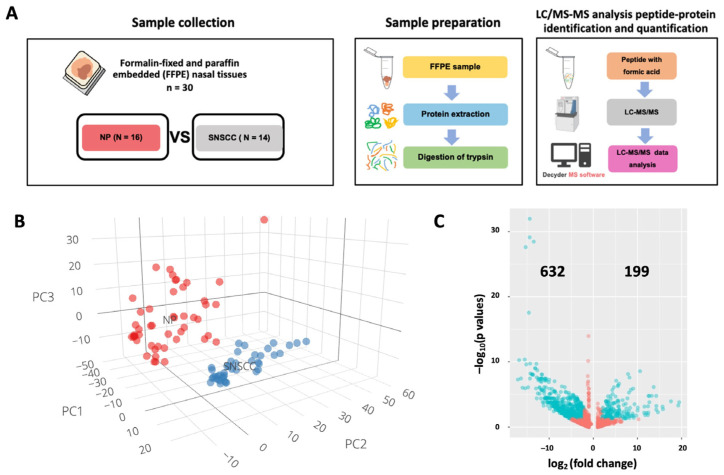
Proteomic profile of NP and SNSCC. (**A**) A total of 30 FFPE samples of NP (N = 16) and SNSCC (N = 14) were used. Proteins were extracted and digested from the samples. Peptides were subjected to LC–MS/MS technology. Relative protein quantification from NP and SNSCC was subjected to Decyder MS analysis and 2324 proteins were detected and quantified. (**B**) PCA analysis of the 30 individual nasal tissue samples with three dependent experiments (NP: 48 datasets, SNSCC: 42 datasets) using the intensity of each protein’s dataset. The NP group (indicated by red dots) was grouped distinctly from the SNSCC group (indicated by blue dots). (**C**) The volcano plot represents differentially expressed proteins (NP vs. SNSCC). Statistical T-tests with fold change ≥±2 and *p* values < 0.05 were considered as a differential expression protein. Red and green dots represent differential expression proteins and non-differential expression proteins, respectively.

To analyze the proteome data of SNSCC by using a mass spectrometry (MS)-based label-free quantification method, we extracted and digested the whole protein from the formalin-fixed paraffin-embedded (FFPE) tissues of 16 NP and 14 SNSCC samples collected in Srinagarind Hospital, Khon Kean University (Figure 1A). The characterization of NP and SNSCC is shown in Table S1. There was a significant difference in age between the two groups. The digested peptide was injected into liquid chromatography–tandem mass spectrometry (LC–MS/MS) and quantified by DecyderMS. The total protein expression profile is shown in Table S2. To investigate the overall differences and similarities of the protein expression profiles between the NP and SNSCC groups, principal component analysis (PCA) was constructed by R programming version 4.3.3. The data from nasal tissues could be clustered distinctly into two groups, indicating that protein signatures can discriminate between NP and SNSCC (Figure 1B),which helped obtain differential expression proteins of SNSCC versus NP for further analysis. From the results, 831 significantly differentially expressed proteins were identified in SNSCC (Figure 1C). Among them, 199 and 632 were up- and down-regulated in the SNSCC, respectively.

### 2.2. Machine Learning for Biomarker Discovery

In this study, we used support vector machine (SVM), logistic regression (LR), random forest (RF), and gradient boost (GB) classifiers to discover a protein that can be a potential tumor-associated marker for SNSCC patients. To evaluate the performance of these models, the dataset was divided into groups, each containing three technical replicates. Leave-one-group-out cross-validation (LOGO-CV) was performed on the entire dataset to assess overall performance and eliminate information leakage from three replicates. The average accuracy across the LOGO-CV was over 70%, indicating consistent and reliable performance in distinguishing SNSCC from NP (Table 1).

Additionally, the dataset was split into a training set (80%) and a validation set (20%) to evaluate model performance on unseen data. The prediction results on the validation set are presented in Table 1 and Supplementary Table S3. Based on the performance for prediction of four models, the RF model showed the best SNSCC prediction performance (accuracy: 94%, precision: 92%, sensitivity: 100%, and precision: 83%). Three models (RF, SVM, and LR) showed more than 70% accuracy, precision, sensitivity, and specificity, indicating they are robust and reliable for classifying SNSCC and NP cases. 

Although the GB model showed a sensitivity of 67%, it achieved a specificity and precision of 100%, indicating its high effectiveness in minimizing false positives. However, its lower sensitivity suggested a reduced ability to identify all true positive cases compared to the other models.

**Figure 2:**
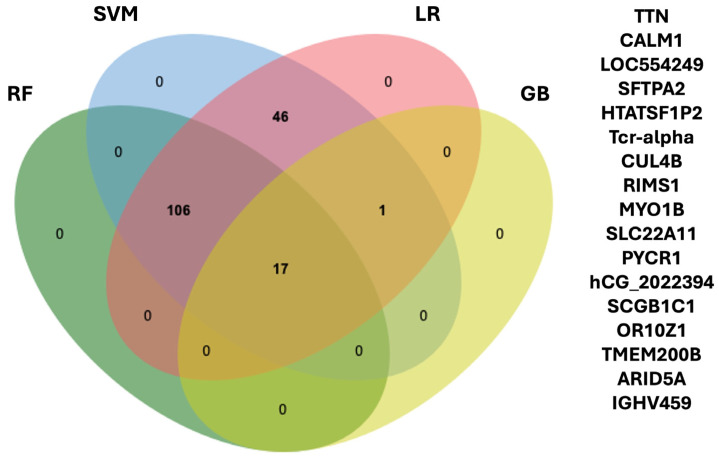
Venn diagram of the proteins the common feature proteins in four machine learning models. A list of 17 common protein panels is presented in right picture.

To select the potential tumor-associated marker for SNSCC, the intersection of feature proteins from each model was used (Table S4). We found that 17 proteins were common in all models, suggesting that there could be a potential tumor-associated marker for SNSCC (Figure 2). Therefore, 17 protein panels could potentially be used as a tumor marker for SNSCC.

### 2.3. Selection of Candidate Biomarkers

**Figure 3:**
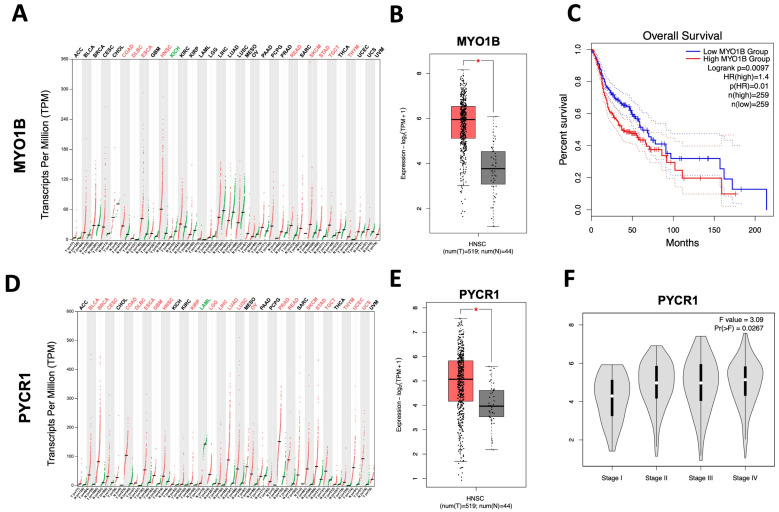
Characterization of MYO1B and PYCR1 expression from the GEPIA2. Pan-cancer analysis of transcriptomic profiles. Red and green letters represent significantly upregulated and downregulated expressions, respectively (**A**,**D**). RNA expression in TCGA-HNSC dataset (**B**,**E**), stages plot (**F**), and its role in the prognosis of HNSC (**C**). * *p* < 0.01.

According to common feature proteins, five criteria were used to select candidate tumor-associated markers for further validation experiments in SNSCC and NP tissues. These included a review of the literature, pan-cancer expression levels, gene expression level in The Cancer Genome Atlas Head-Neck Squamous Cell Carcinoma (TCGA-HNSC) dataset, stage plot, and survival rates using GEPIA2 database. Notably, the genes MYO1B and PYCR1 were significantly upregulated in various cancer types, especially the HNSC dataset, when compared to normal tissues (Figure 3A,B,D,E). Based on the expression levels of MYO1B, we divided the cancer cases into high-expression and low-expression groups and investigated the survival rate of patients with the HNSC dataset. High expression of MYO1B was associated with a poor prognosis for overall survival for HNSC (*p* = 0.0097; Figure 3C). Stage plotting shows that the expression of PYCR1 genes increased with the continuous progression of HNSC patients (*p* = 0.0267; Figure 3F). Thus, we selected MYO1B and PYCR1 for further validation in SNSCC and NP tissues.

### 2.4. PYCR1 Might Serve as a Tumor-Associated Biomarker for SNSCC

**Figure 4:**
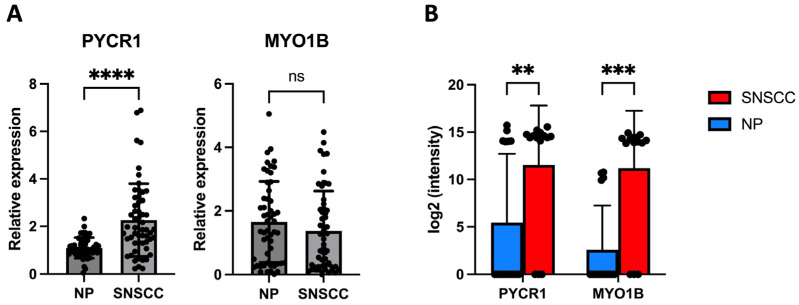
Expression of PYCR1 and MYO1B in SNSCC and NP tissues by qRT-PCR (**A**), proteomic profile (**B**). Results are presented as mean ± SE ** *p* < 0.01, *** *p* < 0.0001, **** *p* < 0.00001, ns = not significant.

To investigate the mRNA expression of PYCR1 in SNSCC and NP tissues, total RNA was extracted from 115 FFPE samples of SNSCC (N = 63) and NP (N = 53). The relative expression levels were examined using RT-qPCR. The expression of PYCR1 mRNA was significantly upregulated in SNSCC compared with NP tissues (two-fold change, *p* < 0.0001; Figure 4A). The overexpression of PYCR1 was further supported by proteomic data, where PYCR1 was found to be significantly upregulated (unadjusted *p* = 0.0099; Figure 4B). After applying the Benjamini–Hochberg (BH) correction for multiple comparisons, the adjusted *p*-value (q-value) increased to 0.062, which exceeded the commonly used significance threshold of 0.05. Despite this, the consistent upregulation of PYCR1 in SNSCC was validated through qRT-PCR, reinforcing its biological relevance as a potential biomarker for SNSCC. In contrast, the expression of MYO1B was not significantly upregulated in SNSCC tissues compared to NP tissues (Figure 4A).

### 2.5. Reviewing of PYCR1 Expression in Different Tumor Tissues and Its Association with Clinicopathological Characteristics in SNSCC Patients

Based on pan-cancer analysis, PYCR1 was consistently expressed across various cancer types. Further review of work in the literature examined the role of PYCR1 in different types of cancer. Higher PYCR1 expression was associated with various clinicopathological features, such as metastasis and advanced tumor stage (Table 2). The biological consequences indicated that PYCR1 affected key hallmarks of cancer, including cell proliferation, anti-apoptosis, and metastasis. Moreover, higher PYCR1 was associated with a worse prognosis. These findings suggested that PYCR1 may serve as a potential oncogene, prognostic biomarker, and therapeutic target for various cancers.

**Figure 5:**
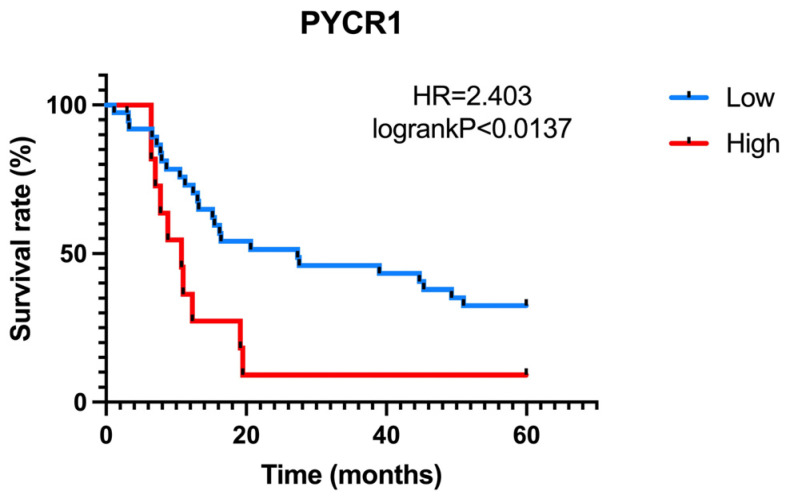
Five-year overall survival rates of PYCR1 expression in SNSCC patients.

As previously indicated, overexpression of PYCR1 was associated with various clinicopathological features and prognostic biomarkers. Therefore, we then examined whether PYCR1 expression was associated with clinicopathological features in SNSCC patients. We divided SNSCC patients into a high PYCR1 expression group (n = 27) and a low PYCR1 expression group (n = 27) according to the median relative expression of SNSCC. However, PYCR1 expression was not found to be associated with age, gender, cell differentiation, sub-type, and invasion (Table S5). To evaluate the prognostic significance of PYCR1 in SNSCC, the R software version 4.3.3 package maxstat was used to determine the optimal cutoff. A Kaplan–Meier analysis with a log-rank test was conducted based on this optimal cutoff, which corresponded to a 4.45-fold change in PYCR1 expression. The results revealed that high PYCR1 expression was significantly associated with poor overall survival compared to low expression (low expression vs. high expression = 27.30 months vs. 10.74 months, HR = 2.40, *p* = 0.0137; Figure 5).

## 3. Discussion

In this study, we integrated proteomic profiles and ML algorithms of SNSCC and NP for a robust classification of these diagnostically challenging tumors. The PCA analysis and volcano plot show different molecularly expressed proteins between NP and SNSCC. Recently, a comprehensive characterization study has been published to identify the signature marker of sinonasal cancer based on epigenetic data [19]. Protein alteration, methylation, and genetic mutations could be identified as potential clinical biomarkers.

Recently, ML has been applied in the field of proteomic analysis to identify important factors in cancer [20]. A proteomic-based ML algorithm can serve as a robust tool for classifying prostate cancer from serum and urine samples, which have more than 80% sensitivity and specificity [21]. The dysregulation of proteins allows us to identify the potential classifiers for differentiating between SNSCC and NP using an ML model. In these studies, we demonstrated that the proteomic-based ML classification algorithm can differentiate between SNSCC and NP with more than 70% accuracy. We also found that RF had the highest performance of the ML models tested. Thus, proteomic-based ML classification algorithms can help clinicians and scientists identify SNSCC. However, the ML classification algorithm was not hyperparameter-tuned, which may contribute to suboptimal performance on new datasets [22].

While our proteomic-based ML algorithm model shows promising results, it has limitations that should be noted. Firstly, the significant age difference between the NP and SNSCC groups may influence the study results, as age is a known risk factor for cancer due to increased genetic mutations and environmental exposures. This difference could affect biomarker identification, as proteomic profiles may reflect age-related changes rather than disease-specific differences. To address this limitation, future studies should validate biomarkers in age-matched cohorts and consider statistical adjustments for age to separate its effects from disease-specific proteomic changes. Secondly, the small sample size of the test set (n = 18) increases variability and uncertainty in performance metrics, as evidenced by wide 95% confidence intervals (95% CIs). Larger datasets and external validation are needed for more reliable, generalizable results and clinical applicability. Thirdly, the application of FFPE-based proteomics methodologies is promising for biomarker discovery. However, FFPE-based proteomics is challenging due to sample quantities, formalin-induced cross-links, and low-abundance protein identification [23,24]. Our dataset utilized 30 individual nasal tissue samples across three dependent experiments, which helped to improve model robustness and account for a broader range of data variations to address the challenges associated with FFPE-based proteomics.

To date, there is a lack of comprehensive data on SNSCC patients due to the tumor rarity. Previous studies have mainly focused on mutations as biomarkers for SNSCC [4,25]. Since SNSCC is a subset of head and neck cancer, we applied the comprehensive TCGA database, mainly in the HNSC dataset, to identify potential tumor-associated markers. Our analysis highlighted the dysregulation of both MYO1B and PYCR1 in different cancer types, especially in the HNSC dataset. Specifically, the expression of MYO1B has been found in various cancer types, such as colorectal cancer and cervical cancer, where it contributes to cell migration, invasion, and metastasis [26,27,28]. This biological consequence was associated with the regulation of the actin cytoskeleton and glycolysis. However, the function of MYO1B in SNSCC is currently unclear. Our RT-qPCR analysis shows that MYO1B mRNA levels were not significantly different between SNSCC and NP tissues. However, proteomic analysis shows that MYO1B levels were significantly upregulated in SNSCC compared to NP tissues. Although there was no correlation between MYO1B mRNA and protein levels, post-transcriptional and post-translational mechanisms may play a key role in regulating MYO1B expression in SNSCC. Epigenetic alteration of miR-145-3p and miR-363 was found to control the expression of the MYO1B gene in head and neck squamous cell carcinoma, which, in turn, led to increased migration and invasion of cancer cells [29,30].

PYCR1 is an enzyme that plays a crucial role in proline biosynthesis. It converts pyrroline-5-carboxylate to proline, an important mechanism for cellular metabolism, stress response, and protein synthesis [31]. Works in the literature and pan-cancer analyses indicate that PYCR1 is commonly upregulated in various cancers, including kidney adenocarcinoma, gastric cancer, lung cancer, pancreatic ductal adenocarcinoma, renal cell carcinoma, breast cancer, and hepatocellular carcinoma. In agreement with previous studies, it was indicated that PYCR1 was the most frequently overexpressed metabolic gene across pan-cancer [31,32]. Silencing of PYCR1 could inhibit cell proliferation, and invasion and enhance the chemosensitivity to doxorubicin in breast cancer cell lines [17]. Additionally, epigenetic alteration of miR-488 was found to negatively regulate PYCR1 expression, leading to inhibition of cell proliferation and tumorigenesis in non-small cell lung [33]. The downstream effects of PYCR1 included the induction of cell proliferation and migration via JAK–STAT3, PI3K/Akt, and Akt–mTOR pathways [13,15,34]. Overall findings indicate that PYCR1 plays an important role in tumor initiation and progression. However, the expression of PYCR1 in SNSCC is still unclear. To the best of our knowledge, our study is the first that integrated proteomic analysis with ML to identify tumor-associated markers and found that the PYCR1 protein was used as a common feature protein in four ML models. Moreover, we confirmed the expression of the PYCR1 gene by using RT-qPCR. PYCR1 mRNA was highly expressed in SNSCC compared with NP tissues, consistent with the findings in the TCGA with head and neck squamous cell carcinoma database. It is confirmed that PYCR1 is significantly overexpressed in SNSCC, suggesting that it plays an important role in tumorigenesis and could be a promising tumor-associated marker in SNSCC. In future studies, PYCR1 could be applied in immunohistochemistry (IHC) to enhance its clinical utility and improve accessibility in routine diagnostics and prognostic evaluations. Additionally, integrating PYCR1 with other biomarkers, such as EGFR mutations, which are used to predict prognosis in SNSCC, could provide a more comprehensive prognostic tool. Previous research shows that higher PYCR1 expression is associated with a worse prognosis in different cancers [10,11,12,13,14,15,16,17,18], which is in agreement with our result that shows high expression of PYCR1 is significantly associated with poor prognosis of SNSCC. Consequently, this finding shows that high expression of PYCR1 can be used as a tumor-associated biomarker to predict the prognosis of SNSCC. However, further studies are needed to elucidate the underlying mechanisms of PYCR1 in SNSCC.

## 4. Materials and Methods

### 4.1. Sample Collection

Left-over FFPE specimens of SNSCC (n = 62) and NP (n = 53) were retrospectively identified from surgical pathology records databases at Srinagarind Hospital, Khon Kaen University, Khon Kaen, Thailand. NP, benign growths with chronic inflammation, was used as nasal tissue control. The sample size (n = 55) was calculated based on the case-control study of Lareo et al., 1992 [35]. All experiments were performed in accordance with the approved guidelines of the Khon Kaen University Ethics Committee for Human Research based on the Declaration of Helsinki and the ICH Good Clinical Practice Guidelines (HE611288, 19 June 2018 and HE671297, 20 May 2024). Due to the retrospective nature of the study, The Khon Kaen University Ethics Committee for Human Research waived the need of obtaining informed consent.

### 4.2. Trypsin-Digested Peptides, LC–MS/MS, and Data Analysis

A total of 30 FFPE samples of NP (n = 16) and SNSCC (n = 14) were sectioned onto tissue slides. The tissue slides were deparaffinized using xylene. Protein extraction was performed on the tumor tissue samples using Qproteome FFPE extraction kit (Qiagen, Hilden, Germany), according to the manufacturer’s instructions. The protein samples were stored at −20 °C until use. To purify the protein prior to liquid chromatography–mass spectrometry (LC–MS/MS) analysis, 750 µL of chilled acetic acid was added to the protein samples, which were then incubated overnight at −20 °C. The protein pellets were collected by centrifugation at 9000× *g* for 15 min and resuspended in 10 mM NH_4_HCO_3_. The protein quantity was measured using Lowry’s method.

For trypsin-digested peptides, 4 µg of each extracted protein sample was prepared and added into a 1.5 mL tube, then dried using a Speed Vac and resuspended with 5 µL of 10 mM NH_4_HCO_3_. Twenty microliters of 5 mM DTT/10 mM NH_4_HCO_3_ were added to each sample tube and incubated at 56° C for 1 h. Next, 20 µL of 15 mM IAA/10 mM NH_4_HCO_3_ were added and incubated in the dark at room temperature for 1 h. A total of 4 µL of 50 ng/µL trypsin in 10 mM NH_4_HCO_3_ were added and incubated overnight at 37 °C. Finally, the digested samples were dried using Speed Vac, and the peptides were resuspended with 0.1% formic acid.

The trypsin-digested peptides were injected into the LC–MS/MS analyzer with three dependent experiments (Hybrid quadrupole Q-TOF impact II™, Bruker Daltonics, Billerica, MA, USA). The peptides were separated by using the Ultimate3000 Nano/Capillary LC System (Thermo Scientific, Waltham, MA, USA) coupled with Nano-captive spray ion source. LC–MS/MS analysis and protein quantification were performed as previously described [36]. Briefly, LC–MS/MS raw data files were analyzed using DeCyderMS 2.0 differential analysis software (GE Healthcare Life Science, Amersham, UK) and subjected to Mascot software version 2.7.0 (accessed in May 2020). (Matrix Science, London, UK) to search the proteins name and proteins score based on the NCBI database with the following parameter; Homo sapiens (AA) database; trypsin enzyme; allowed up to three missed cleavage; carbamidomethyl (C) as fixed modification and oxidation (M) as variable modification; peptide charge state of +1, +2, +3; ESI-QUAD-TOF instrument type and report 1000 top hits. Mascot dat. files were imported to the Decyder PepMatch 2.0 software (accessed in May 2020). The MS data were exported to the text file.

### 4.3. Principal Component Analysis and Identification of Differentially Expressed Genes

For PCA analysis, 30 individual nasal tissue samples with three dependent experiments (total 90 datasets; NP = 48 datasets; SNSCC = 42 datasets) using the intensity of each protein’s dataset were constructed by R programming with ggplot2 and plotly packages. To identify the differentially expressed proteins, the relative protein expression values were compared between the NP and SNSCC groups. The proteins were differentially expressed if the relative protein expressions were >±2 ratio of log2 (SNSCC/NP) intensity, with a *p*-value < 0.05, which was statistically analyzed by a paired *t*-test. For multiple comparisons, the Benjamini–Hochberg (BH) procedure was applied to control the false discovery rate (FDR). The adjusted *p*-values, or q-values, were reported. We used a volcano plot to display the differentially expressed proteins by using R programming with ggplot2 package, where the *x*-axis represents the log2-based fold change, and the *y*-axis represents the negative log_10_ of the *p*-value calculated from the two-tailed *t*-test.

### 4.4. Machine Learning Models

**Figure 6:**
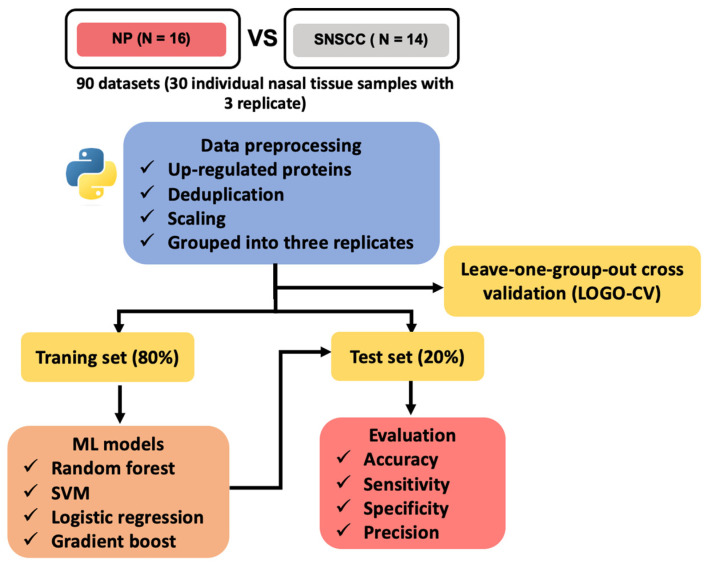
Workflow of machine learning models used to predict SNSCC. ML machine learning; SVM support vector machine.

The workflow of this study is shown in Figure 6. Support vector machine (SVM), logistic regression (LR), random forest (RF), and gradient boost (GB) classifiers were developed to predict tumor markers based on proteomic profiles of SNSCC and NP, using Python libraries Pandas, NumPy, Matplotlib, and Scikit-learn. The differentially up-regulated proteins in SNSCC were used as an input dataset. Ninety datasets of proteomic profiles were deduplicated, scaled, and grouped into three replicates. LOGO-CV was applied to the entire dataset and aggregated predictions and true labels for all iterations of LOGO-CV. Subsequently, performance metrics, such as accuracy, were then averaged across these iterations.

To extract the featured protein from each ML, the dataset was randomly split into a training set (n = 72) and a validation set (n = 18), containing 80% and 20%, respectively. The training set was used to train four ML models, and their performance was evaluated on the validation set using a confusion matrix such as accuracy, sensitivity, specificity, and precision. Feature proteins for each model were identified during this process. Ninety-five percent CIs were calculated using the epiR package. The intersection of feature proteins was selected using the jvenn online tool (http://jvenn.toulouse.inrae.fr/app/example.html, accessed on 10 February 2024).

### 4.5. In Silico Analysis of PYCR1 and MYO1B Gene Expression Based on Pan-Cancer Database

The pan-cancer gene expression data, HNSC-TCGA dataset with stage plot and survival rate were analyzed by employing GEPIA2 (http://gepia2.cancer-pku.cn/#index, accessed on 24 February 2024) [37], a web server for gene expression analysis based on the RNA-seq data of 9736 tumors and 8587 normal samples from the The Cancer Genome Atlas (TCGA). 

### 4.6. Relative Gene Expression by qRT-PCR

A total of 115 FFPE samples of NP (N = 53) and SNSCC (N = 62) were used for validation. Total RNA was extracted using a High Pure RNA Paraffin Kit (Roch, Mannheim, Germany), according to the manufacturer’s instructions. Total RNA was synthesized to cDNA according to the manufacturer’s protocol (RevertAid H minus First Strand cDNA Synthesis Kit, ThermoFisher Scientifics, Waltham, MA, USA). To determine relative gene expression, gene expression was analyzed by qRT-PCR using SsoAdvancedTM SYBR^®^ Green SuperMix (Bio-Rad, Hercules, CA, USA) in QuantStudio™ 6 Flex Real-Time PCR (Applied Biosystems, Foster City, CA, USA). GAPDH was used as an internal control. The relative expression level of targeted mRNA was determined using the comparative CT method (2^−ΔΔCT^ method). The primers used in the present study are listed in Table 3.

### 4.7. Statistical Analysis

GraphPad Prism 9 software (GraphPad Software Inc., San Diego, CA, USA) was used for data analysis. The relative gene expression data were analyzed using nonparametric tests with Mann–Whitney test. The optimal cutoff value for categorizing patients into high and low PYCR1 expression groups was determined using the maxstat R package. Survival analysis of PYCR1 expression in SNSCC (n = 49) patients was analyzed using the Kaplan–Meier method with a log-rank test. Fisher exact test was used to evaluate the relationship between PYCR1 group and clinicopathological features. All statistical tests were two-sided. *p*-value of <0.05 was considered statistically significant.

## 5. Conclusions

Our study utilized proteomic and ML approaches to identify potential biomarkers for sinonasal squamous cell carcinoma (SNSCC). Seventeen feature proteins were found in all models. PYCR1 was validated as a significant SNSCC marker through RT-qPCR, and its high expression correlated with poor overall patient survival, suggesting PYCR1 could serve as a tumor-associated prognostic biomarker for SNSCC.

## Figures and Tables

**Table 1:** Performance of the four machine learning classification models.

Models	LOGO-CV (95%Cl)	Accuracy (95%Cl)	Sensitivity (95%Cl)	Specificity (95%Cl)	Precision (95%Cl)
Random forest (RF)	0.97 (0.91, 0.99)	0.94 (0.73, 1.00)	1.00 (0.74, 1.00)	0.83 (0.36, 1.00)	0.92 (0.64, 1.00)
Support vector machine (SVM)	0.92 (0.85, 0.97)	0.89 (0.65, 0.99)	1.00 (0.74, 1.00)	0.67 (0.22, 0.96)	0.86 (0.57, 0.98)
Logistic regression (LR)	0.92 (0.85, 0.97)	0.89 (0.65, 0.99)	1.00 (0.74, 1.00)	0.67 (0.22, 0.96)	0.86 (0.57, 0.98)
Gradient boost (GB)	0.73 (0.63, 0.82)	0.78 (0.52, 0.94)	0.67 (0.35, 0.90)	1.00 (0.54, 1.00)	1.00 (0.63, 1.00)

**Table 2:** Characteristics of PYCR1 in different types of cancer.

Cancer Type	Year	Molecular Mechanism Findings	Association with Clinical Features	Association with Prognosis/Diagnostic	Ref.
Adenocarcinoma of the kidney	2021	-	High PYCR1 expression were associated with high histologic grade, advanced clinical stage, and presence of metastasis.	Increased PYCR1 expression was associated with a worse prognosis.	[10]
Gastric cancer	2020	Silencing of PYCR1 inhibited cell proliferation and induced apoptosis; PI3K/Akt pathway can affect proline metabolism via PYCR1.	High PYCR1 expression was associated advanced stage, histologic type, and high Ki-67.	Increased PYCR1 expression was associated with a worse prognosis.	[11]
Non-small cell lung cancer	2018	Silencing of PYCR1 inhibited cell proliferation, induced cell cycle arrest, and increased apoptosis.	-	Increased PYCR1 expression was associated with poor prognosis.	[12]
Lung cancer	2023	PYCR1 induced cell proliferative, migration, and invasion through the JAK–STAT3 signaling pathway via PRODH-dependent glutamine synthesize.	High PYCR1 expression was associated with advanced stage.	Increased PYCR1 expression was associated with poor prognosis; PYCR1 secretion in serum may use as a diagnostic marker.	[13]
Pancreatic ductal adenocarcinoma	2022	Silencing of PYCR1 inhibited cell proliferation and induces apoptosis.	-	Increased PYCR1 expression was associated with a worse prognosis.	[14]
Gastric cancer	2024	PYCR1 induced cell proliferation and metastasis and suppressed the apoptosis via the PI3K/AKT signaling.	-	Increased PYCR1 expression was associated with unfavorable prognosis.	[15]
Renal cell carcinoma	2019	-	High PYCR1 expression was associated with metastasis.	Increased PYCR1 expression was associated with poor prognosis.	[16]
Breast cancer	2017	Silencing of PYCR1 inhibited cell proliferation, invasion and enhanced the chemosensitivity to doxorubicin.	High PYCR1 expression were associated with larger tumor size, higher tumor grade, and more invasive molecular subtypes.	Increased PYCR1 expression was associated with poor prognosis.	[17]
Hepatocellular carcinoma	2021	Silencing PYCR1 inhibited cell proliferation, invasion, epithelial–mesenchymal transition, and metastasis	High PYCR1 expression were associated with female sex, higher alpha-fetoprotein levels, advanced clinical stage, and younger age (<45 years)	Increased PYCR1 expression was associated with poor prognosis	[18]

**Table 3:** List of primers.

Gene	Forward (5′-3′)	Reverse (5′-3′)	Ref.
*GAPDH*	TCATCAGCAATGCCTCCTGCA	TGGGTGGCAGTGATGGCA	[38]
*PYCR1*	GTGGTTACTGTGGGTGGAATA	CAGATGCCCTCCAAGATGTG	[39]
*MYO1B*	GGTCTGGTGTGGAGGTCCTA	CGTTGCTTCCTCAGGTCTTC	[26]

## Data Availability

The LC–MS/MS raw data have been deposited in the MassIVE database with accession number MSV000095570. The analysis methodology associated with this article is available on GitHub (https://github.com/watcharapong2538/ML_proteomic_SNSCC, accessed on 22 July 2024).

## Supplementary Materials

The following supporting information can be downloaded at: https://www.mdpi.com/article/10.3390/ijms252413234/s1.
