# Supplementary material for: Integrated Proteomics and Machine Learning Approach Reveals PYCR1 as a Novel Biomarker to Predict Prognosis of Sinonasal Squamous Cell Carcinoma
Source: Int J Mol Sci. 2024 Dec 10;25(24):13234. doi: 10.3390/ijms252413234 (PMC11675701; doi:10.3390/ijms252413234)
Supplement: Supplementary file 1 [file ijms-25-13234-s001.zip › Table S1.pdf]

Table S1. Characterization of Nasal Polyps (NP) and Sinonasal Squamous Cell Carcinoma (SNSCC) in Proteomic Study

Models	NP (n = 16)	SNSCC (n = 14)	p-value	Statistical Tests
Age (mean ± SD)	40.75 ± 20.59	59.28 ± 11.22	0.0048	T-test
Sex (Male)	12	11	>0.9999	Fisher exact test
Sex (Female)	4	3		
