# Supplementary material for: Integrated Proteomics and Machine Learning Approach Reveals PYCR1 as a Novel Biomarker to Predict Prognosis of Sinonasal Squamous Cell Carcinoma
Source: Int J Mol Sci. 2024 Dec 10;25(24):13234. doi: 10.3390/ijms252413234 (PMC11675701; doi:10.3390/ijms252413234)
Supplement: Supplementary file 1 [file ijms-25-13234-s001.zip › Table S3.pdf]

Table S3. Other performance parameter of the four machine learning classification models.

Models	R-square	F1-score	AUROC	AUPRC
Random forest (RF)	0.94	0.96	0.99	0.99
Support vector machine (SVM)	0.89	0.92	0.85	0.87
Logistic regression (LR)	0.89	0.92	0.86	0.90
Gradient boost (GB)	0.78	0.80	0.95	0.97
