# Supplementary material for: Integrated Proteomics and Machine Learning Approach Reveals PYCR1 as a Novel Biomarker to Predict Prognosis of Sinonasal Squamous Cell Carcinoma
Source: Int J Mol Sci. 2024 Dec 10;25(24):13234. doi: 10.3390/ijms252413234 (PMC11675701; doi:10.3390/ijms252413234)
Supplement: Supplementary file 1 [file ijms-25-13234-s001.zip › Table S5.pdf]

Table S5. PYCR1 expression association with clinicopathological characteristics in SNSCC patients.

**Clinical characteristics**		**PYCR low (n=27)**	**PYCR high (n=27)**	**Fisher's exact test (p-value)**
Gender	Male	21	7	0.371
	Female	6	10	
Age	≤55 years	11	9	0.778
	>55 years	16	18	
Cell differentiation	Well differentiated	4	3	0.651
	Moderate-poor-undifferentiated	6	9	
Sub-type	Keratinizing	2	2	0.999
	Nonkeratinizing	9	9	
Invasion	Invasion	11	9	0.135
	Non-invasion	16	18	
